# 

**Published:** 1885-08

**Authors:** 


					﻿Wh6le Number,
CCLXXIX.
AUGUST, 1885.
Number i.
Volume XXV.
The
BUFFALO
Medical Surgical Jurnal
EDITORS:
• r HdsNiLdxjEmoi',. m. n.
A. R. DAVIDSON, M. ».
Published by the Medical Journal Association.
No. 8 WEST CHIPPEWA ST.
Entered at the Post-Office at Buffalo, N. Y., as Second-class Mail Matter-
				

